# Facile Synthesis, Characterization, and Antimicrobial Assessment of a Silver/Montmorillonite Nanocomposite as an Effective Antiseptic against Foodborne Pathogens for Promising Food Protection

**DOI:** 10.3390/molecules28093699

**Published:** 2023-04-25

**Authors:** Mohsen M. El-Sherbiny, Reny P. Devassy, Mohamed E. El-Hefnawy, Soha T. Al-Goul, Mohammed I. Orif, Mohamed H. El-Newehy

**Affiliations:** 1 Department of Marine Biology, Faculty of Marine Sciences, King Abdulaziz University, Jeddah 21589, Saudi Arabia; 2 Department of Chemistry, Rabigh College of Sciences and Arts, King Abdulaziz University, Jeddah 21589, Saudi Arabia; 3 Department of Marine Chemistry, Faculty of Marine Sciences, King Abdulaziz University, Jeddah 21589, Saudi Arabia; 4 Department of Chemistry, Faculty of Science, Tanta University, Tanta 31527, Egypt; 5 Department of Chemistry, College of Science, King Saud University, Riyadh 11451, Saudi Arabia

**Keywords:** foodborne bacteria, food packaging, AgNPs/MMT nanocomposite, XRD, antibacterial activities, MIC

## Abstract

Foodborne pathogens can have devastating repercussions and significantly threaten public health. Therefore, it is indeed essential to guarantee the sustainability of our food production. Food preservation and storage using nanocomposites is a promising strategy. Accordingly, the present research’s objectives were to identify and isolate a few foodborne pathogens from food products, (ii) synthesize and characterize silver nanoparticles (AgNPs) using wet chemical reduction into the lamellar space layer of montmorillonite (MMT), and (iii) investigate the antibacterial potential of the AgNPs/MMT nanocomposite versus isolated strains of bacteria. Six bacterial species, including *Escherichia coli*, *Salmonella* spp., *Pseudomonas aeruginosa*, *Staphylococcus aureus*, *Listeria monocytogenes*, and *Bacillus cereus* were isolated from some food products (meat, fish, cheese, and vegetables). The Ag/MMT nanocomposite was synthesized and characterized using UV–visible spectroscopy, transmission electron microscopy, particle size analyzer, zeta potential, X-ray diffraction (XRD), and scanning electron microscopy with dispersive energy X-ray (EDX). The antibacterial effectiveness of the AgNPs/MMT nanocomposite further investigated distinct bacterial species using a zone of inhibition assay and microtiter-based methods. Nanoparticles with a narrow dimension range of 12 to 30 nm were identified using TEM analysis. The SEM was employed to view the sizeable flakes of the AgNPs/MMT. At 416 nm, the most excellent UV absorption was measured. Four silver metallic diffraction peaks were found in the XRD pattern during the study, and the EDX spectrum revealed a strong signal attributed to Ag nanocrystals. AgNPs/MMT figured out the powerful antibacterial action. The AgNPs/MMT nanocomposite confirmed outstanding minimum inhibitory concentration (MIC) and minimum bactericidal concentration (MBC) against six isolates of foodborne pathogens, ranging from 15 to 75 µg/mL, respectively. The AgNPs/MMT’s antibacterial potential against gram-negative bacteria was noticeably better than gram-positive bacteria. Therefore, the AgNPs/MMT nanocomposite has the potential to be used as a reliable deactivator in food processing and preservation to protect against foodborne pathogenic bacteria. This suggests that the nanocomposite may be effective at inhibiting the growth and proliferation of harmful bacteria in food, which could help to reduce the risk of foodborne illness.

## 1. Introduction

Food spoiling is the process of infecting food in such a way as to reduce its nutritive benefits, firmness, and texture, as well as to facilitate the development of harmful microorganisms that seem to be present in food and make it unsuitable to consume [1,2]. Foodborne disorders, including spoilage pathogens and foodborne poisoning, can be driven on by packaged foods infected with microbial pathogens (bacteria, parasites, and viruses). These illnesses can range from mild to severe and can cause symptoms such as nausea, vomiting, diarrhea, and fever. To prevent food spoilage and reduce the risk of foodborne illness, it is essential to handle and store food properly, follow safe food preparation practices, and be aware of the potential dangers of consuming certain foods that threaten human health severely [3]. The pathogen’s cells cause serious illness once they spread throughout an infected person after entering the body through contaminated food. At the same time, food poisoning arises after developing dangerous pathogenic species in food and the emission of toxins consumed by the infected individual [3,4]. According to the official World Health Organization (WHO) report, diarrheal diseases lead to 550 million cases and 230,000 deaths annually [5]. 

To heighten food integrity, prolong shelf life, and avoid or postpone deterioration, antimicrobial food packaging, such as special packaging, that distributes potent biocidal compounds is created quicker because of the importance of eliminating foodborne pathogens [2]. Consequently, innovative packaging material treatments with better sustainability are now being investigated as the requirement of the day to address the steadily increasing needs and requirements for food quality and safety and to reduce bacterial spoilage [6]. The antibacterial effect can be produced by scattering the preserving material about the meal or straight inside it [1]. Organic acids, enzymes, and polymers constitute the majority of the first category, whereas metallic nanoparticles (NPs) comprise the vast majority of the second. It is interesting to reveal that while metal and organic NPs may survive more demanding processing conditions, organic antimicrobial substances are much less stable at greater temperatures than metal antimicrobial chemicals [7]. 

The antimicrobial nanoparticles’ protective qualities and desirable structural integrity make them particularly fascinating since they reduce harmful microbes’ deterioration and reproduction [8]. Incorporating several antimicrobial agents and exploiting their synergistic antiseptic activity are among the innovative approaches to enhance the efficiency of eradicating microbial pathogens. These treatments may include different processing techniques, preservatives, food packaging, or storage conditions [9]. Food packaging using nanomaterials has given extensive interest because of its excellent alternative approach for decreasing, eliminating, or inhibiting the evolution of harmful and spoiled microbes in packaged foods and prolonging storage life. Further, antimicrobial packaging sheets are created by combining the antimicrobial compounds in polymer matrices to prevent the development of specific microorganisms whose activities would otherwise contaminate the food [10].

Nanotechnology can be utilized to create food packaging, which is divided into two main categories. An example of improved packaging is clay nanocomposites, which mix nanostructures with polymer matrices to improve their oxygen barrier properties. The second example is “active packaging,” in which metallic nanoparticles directly interact with the food or the environment to enhance food safety [11]. Silver nanoparticles (AgNPs), which are among the metallic nanoparticles, have drawn more interest recently because of their distinct physical, biological, and chemical characteristics. Likewise, AgNPs are recognized for having a significant antibacterial action against various pathogens, including bacteria viruses, because of their tiny particle size, outsized surface area, and biocompatibility [12]. AgNPs have also been utilized for decades to protect and manage several chronic diseases, mainly illnesses. They are widely recognized to have potent bactericidal and inhibiting actions and a broad spectrum of antimicrobial properties [13]. Notably, AgNPs were recently documented to have bactericidal activity against some foodborne bacteria such as *E. coli*, *S. aureus* [14], *P. aeruginosa*, *Salmonella enterica*, *Bacillus cereus* [15], and *L. monocytogenes* [16]. Further, AgNPs have significant immunosuppressive effects on HIV-infected cells [17].

Likewise, bulk metallic Ag^+^ ions are a silver source that can synthesize AgNPs through reduction reactions. The high electronegativity of silver makes it a potent oxidizing agent, which allows it to react efficiently with other chemicals and form stable compounds. In addition, silver has low toxicity and is generally considered safe for use in various applications [18]. Similarly, it is possible and feasible to use montmorillonite (MMT) to distribute AgNPs due to its relatively high charge that permits expansion in solution and creates a persistent pseudo-cross-linking network that can stabilize AgNPs [19]. This can enhance the antimicrobial properties of the AgNPs and provide a stable matrix for their delivery in various applications [20,21]. 

The prime purpose of the current investigation is to assess the antibacterial activity of a synthesized AgNPs/MMT nanocomposite against some foodborne pathogens isolated from food product samples. Six foodborne bacterial species were isolated from food products and identified using phenotyping. Likewise, the AgNPs/MMT nanocomposite was synthesized using NaBH_4_ as a reduction agent at room temperature. Additionally, the antibacterial properties of the AgNPs/MMT nanocomposite were deeply explored.

## 2. Results and Discussion

**Figure 1:**
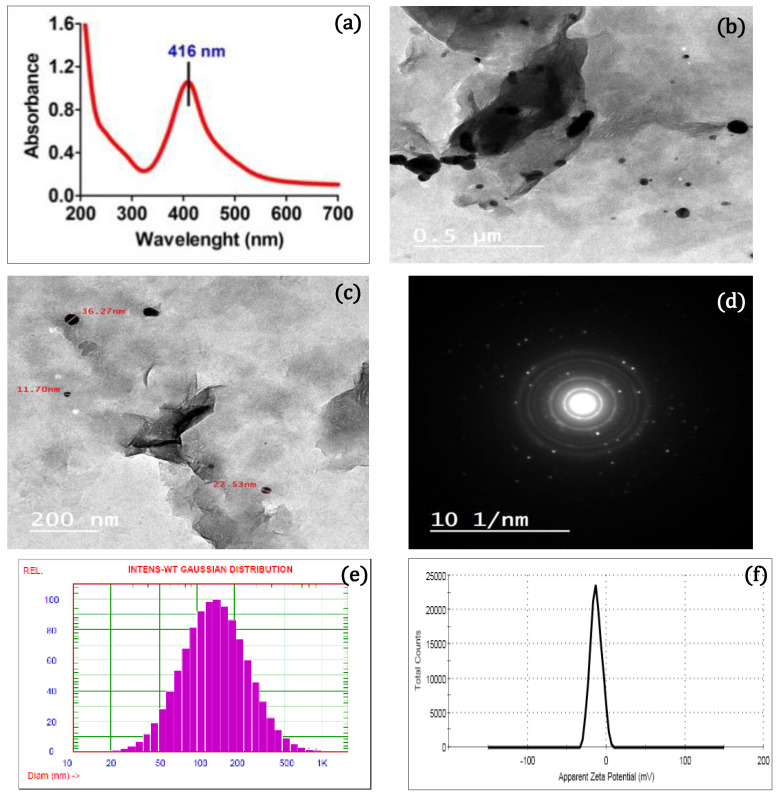
(**a**) UV–Vis absorption spectrum, (**b**) TEM images with 200 nm of magnification, (**c**) TEM images with 0.5 nm of magnification, (**d**) the diffraction rings in a SAED pattern, (**e**) particle size, and (**f**) zeta potential of the AgNPs/MMT nanocomposite.

The wet chemical reduction was employed to compose the AgNPs/MMT nanocomposite in the existence of NaBH_4_ as a powerful reductant for bactericidal studies. The synthesized AgNPs/MMT nanocomposite was analyzed using a range of methodologies comprising UV–Vis absorbance, TEM, zeta potential, particle size, XRD, and SEM. Because of the simultaneous vibration of the metal NPs’ electronegativity in resonance frequency whenever the AgNP amplitude was much smaller than the light beam, which eventually ends in collective dipole oscillation and surface-charged particle polarization, AgNPs have electron density which would sustain spectral response (SPR) uptake band [22]. As a result, using a UV–Vis spectrometer to measure the samples’ UV–Vis absorption spectra is the most practical way to assess the synthesis of AgNPs [23]. The alteration in coloration is the main sign that AgNPs are producing. Likewise, AgNPs have a distinct maximum absorption in the visible light spectrum between 380 and 450 nm, which varies depending on the size, structure, and interactions of the NPs with the substrate, including particle agglomeration [24]. Thus, the prepared nanocomposite was identified to exist in the 200–700 nm range using UV–visible spectroscopy. Throughout our investigation, the change in color intensity to a dark brown, signifying the formation of AgNPs/MMT nanocomposite, was utilized to verify the production and presence of AgNPs in MMT through the lowering impact of NaBH_4_. The SPR bands of AgNPs were discovered at about 416 nm as an absorption peak, as seen in Figure 1a. 

Further size, structure, and dispersion measurements of Ag^+^ in the AgNPs/MMT nanocomposite were undertaken using TEM (Figure 1b,c). In the AgNPs/MMT nanocomposite, AgNPs exhibited a propensity to assemble into greater NPs with dimension distributions of NPs of 12.1–21.6 nm. Additionally, in the AgNPs/MMT nanocomposite, AgNPs with a mean particle size of 11.3 nm were produced. 

The method known as selected area electron diffraction (SAED) is used to identify the crystal structure of various materials. The outcomes of SAED research can reveal details regarding the crystal structure, size, and shape of nanoparticles. If the SAED findings show that the AgNPs were organically crystalline, it suggests that the nanoparticles were made of organic molecules and had a clearly defined crystal structure. To put it another way, the AgNPs had a particular arrangement of atoms and molecules that were repeated throughout the particle. This arrangement was made up of both organic and Ag atoms. AgNPs’ properties, including their stability and reactivity, can be affected by the presence of organic molecules in their crystal structure. The crystalline nature of the AgNPs/MMT was confirmed by the SEAD pattern), which showed the quasi-ring-like diffraction pattern, demonstrating that the polycrystalline structure was formed, and the (110), (111), (211), (220), and (311) rings were indexed to the face-centered cubic (fcc) crystal. The SAED results indicated that the AgNP/MMT nanocomposites were crystalline (Figure 1d). Given that it is much smaller than the typical size of AgNPs created through consistent nucleation and stability in solutions (lower than 25 nm), our data suggest that MMT’s capture of AgNPs was more effective. The existence of a tiny particle of Ag over the MMT surface conforms with the results publicized by Praus et al. [25].

A PSA analyzer was utilized to determine the mean size and distribution of the MMT/Ag nanocomposite. To create liquid formulations for the PSA, 5 mg of powdered sample was combined with the DW for 10 min. Figure 1e,f demonstrates the PSA and zeta potential assessment of the prepared nanocomposite. Following the results, the average AgNPs/MMT particle size was approximately 135.3 nm, and its zeta potential value was −32.13 mv. Yan et al. [26] stated that the MMT’s mean particle sizes were 20.82 nm. Furthermore, according to Gashti et al., the presence of AgNPs on the surface of MMT clay reduced the material’s thermal stability due to the hydroxyl groups in the nanocomposite’s proton delocalization [27].

**Figure 2:**
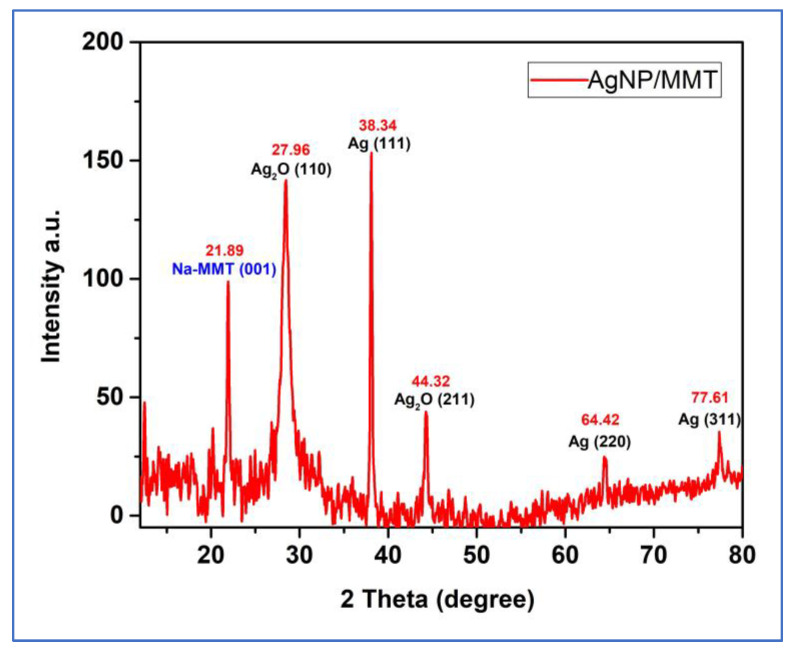
The XRD pattern of the AgNPs/MMT nanocomposite.

XRD is a valuable characterization tool to estimate the size of crystalline nanostructures, establish the crystalline phase, and confirm the creation of AgNPs [28,29]. AgNPs/MMT nanocomposite’s X-ray lattice was analyzed. The XRD characterization of the AgNPs/MMT revealed the crystalline phase of AgNPs. Six different intense peaks in the 2*θ* range of 10–80° were perceived. The diffraction profile had intense peaks at 2*θ* of 21.89°, 27.96°, 38.34°, 44.32°, 64.42°, and 76.61°, corresponding to the (001), (110), (111), (211), (220), and (311) planes. It established the direct production of single-phase and cubic-structure AgNPs (Figure 2 and Table 1). The lattice planes of a crystal structure refer to the planes of atoms that make up the crystal lattice. In the case of silver nanoparticles, the lattice planes can significantly affect their properties, including their bactericidal potential. Silver nanoprisms with (111) lattice planes showed the most significant bactericidal potential compared to other crystallographic plane nanoparticles. This may be because the (111) plane is an exceptionally reactive surface of the silver nanoparticle, which makes it more effective at interacting with bacterial cells and disrupting their function. It is important to note, however, that the specific bactericidal potential of silver nanoparticles can depend on a variety of factors, including the size and shape of the nanoparticles, the type of bacteria being targeted, and the conditions under which the nanoparticles are used.

Further studies would be needed to fully understand the relationship between lattice planes and bactericidal potential in silver nanoparticles [30]. The XRD pattern of the produced AgNP/MMT revealed strong peaks, demonstrating the existence of a clearly defined crystalline structure in the NPs. The crystal structure and orientation of the nanoparticles can be determined from the locations and intensities of the peaks in the XRD pattern. The crystalline phases present in the generated AgNP/MMT may be recognized by comparing the experimental XRD pattern with the identified patterns of other materials [31]. The outcomes verified that the nanocomposite included MMT clay which had Ag ions. Due to the addition of MMT and MMT-Ag, alterations in the relative intensities of the maxima corresponding to the nanocomposite also indicated a considerable structural change. The AgNO_3_ effectively adsorbed on MMT reduced to metallic NPs to produce AgNPs/MMT nanocomposites, of which the antibacterial capacity was assessed, according to the results of the UV–Vis and XRD analyses.

**Table 1:** XRD data of simple peak indexing of the AgNPs/MMT nanocomposite.

Pos. [°2Th.]	Height [cts]	Plane	Specie	References
21.89	97.7	001	NaMMT	[25]
27.96	135.8	110	Ag_2_O	[32]
38.34	151.1	111	Ag	[18,33,34]
44.32	41.8	211	Ag_2_O	[32]
64.42	23.3	220	Ag	[18,33]
77.61	34.01	311	Ag	[18,33]

**Figure 3:**
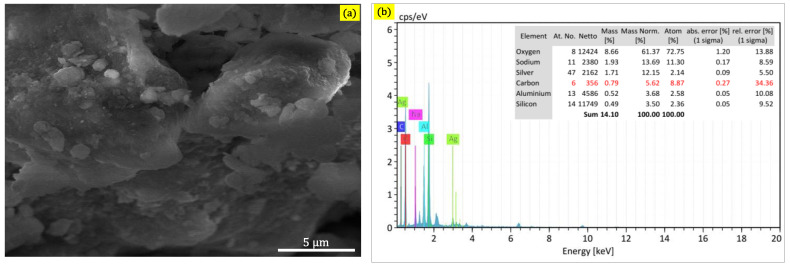
(**a**) FESEM image and (**b**) elemental composition by EDX of the AgNPs/MMT nanocomposite.

The existence of AgNPs on large flakes of MMT clay was remarkable in the micro-FESEM images of the AgNPs/MMT nanocomposite, indicating a connection between two MMT and AgNPs in the nanocomposite interaction. Using FESEM and EDX, the surface structures and elemental constitution of the AgNPs/MMT nanocomposite were analyzed. The surface morphology of the nanocomposite, which is standard for MMT, can be observed in the SEM image to be layered with some sizable flakes. On the AgNPs/MMT nanocomposite, small particles, possibly the Ag^+^, were seen (Figure 3a). 

As depicted in Figure 3b, the EDX spectrum for the AgNPs/MMT nanocomposite proved that there were no impurity signals and that the Ag^+^ elemental constituents were included in the MMT nanocomposite. The findings suggested that silicon, aluminum, and silver are indeed the critical ingredients of the nanocomposite, which has a flaky and multilayer architecture. The nanocomposite’s elemental constitution, as determined by EDX, includes Si (15.7%), Al (5.5%), Na (2.5%), and Ag (8.17%). Thus, the results of the microscopic investigation point to the nanocomposite being a heterogeneous mixture that comprises MMT flakes and AgNPs. Regarding the toxicity results, it can be exhibited that the value of EC50% for AgNPS/MMT nanocomposites was 248%, implying that this nanocomposite is biocompatible. Generally, using bulk metallic Ag^+^ ions and montmorillonite clay as suppliers of AgNPs can offer several advantages, including low toxicity, chemical stability, and strong antimicrobial properties. However, it is crucial to carefully evaluate the potential risks and benefits of any new material or technology before using it in food processing or other applications.

### 2.1. Isolation and Identification of Foodborne Bacterial Isolates from Food Samples

**Figure 4:**
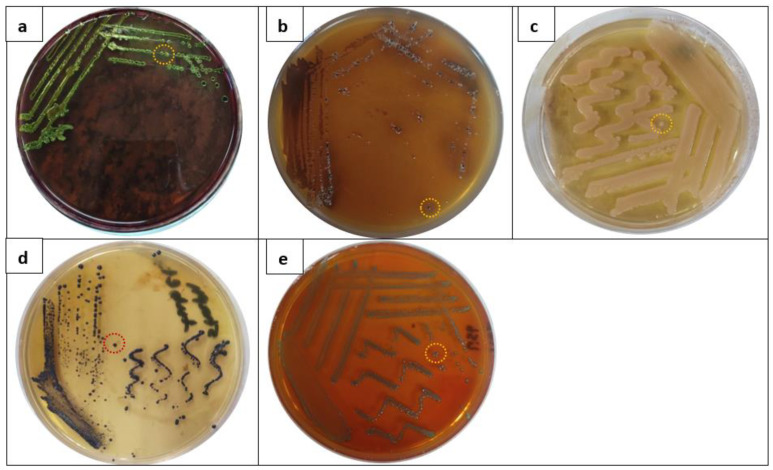
A distinctive feature of standard colony of each bacterial isolate (**a**) *E. coli*, (**b**) *Salmonella* spp., (**c**) *P. aeruginosa*, (**d**) *S. aureus*, and (**e**) *L. monocytogenes* cultivated on selective media.

First, we designed this work to isolate some predominant foodborne bacterial strains from food product samples. Results of the screening procedure of six abundant foodborne bacterial pathogens, including *E. coli, Salmonella* spp., *P. aeruginosa*, *S. aureus*, *L. monocytogenes*, and *B. cereus*, were tabulated in Table 2. Results of collected food products samples exhibited that 71.6% (43/60) of food samples were positive for *E. coli*, followed by salmonella (68.3%), *P. aeruginosa* (51.6%), *S. aureus* (48.3%), *L. monocytogenes* (46.6%), and *B. cereus* (35%). Afterward, based on the morphological appearance of bacterial colonies of each bacterial isolate, a typical single colony of each bacterial isolate was carefully picked up for identification by phenotyping methods (Figure 4). The use of metabolic activities processes and nitrogen sources is essential for classical phenotypic identification. Over the years, researchers have tried a variety of automation-based advancements. One such effort is the Microlog platform station, which uses a microtiter plate to examine a microorganism’s capacity to use various carbon sources. The method tests a pattern of colored wells that functions as the implanted organism’s biochemical fingerprints. In the present research, the Biolog Microstation platform was applied for classifying the bacterial isolates for further investigations (Supplementary Data). 

### 2.2. Antibacterial Evaluation

**Figure 5:**
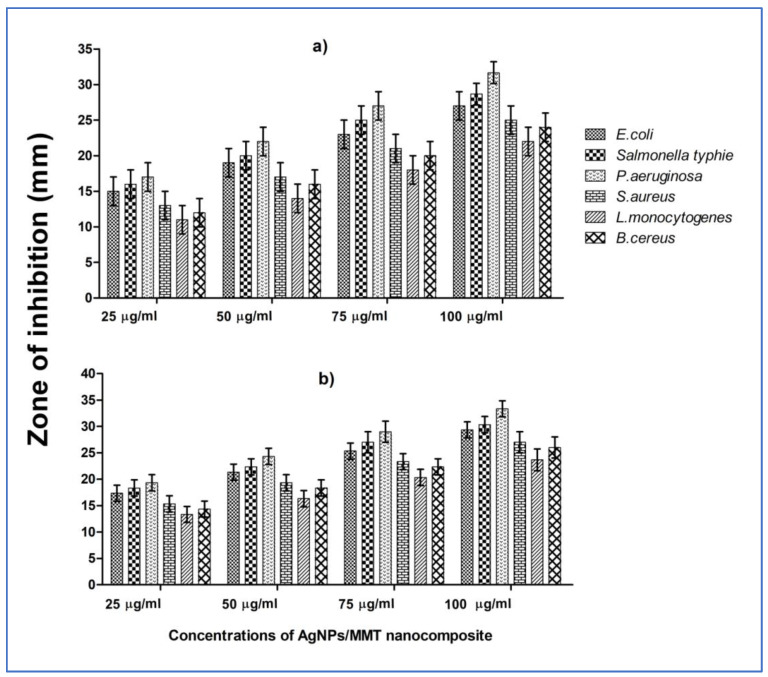
Antibacterial activity and ZOI of the AgNPs/MMT nanocomposite at various concentrations (25–100 µg/mL) using (**a**) disc and (**b**) well diffusion assays toward selected bacterial pathogens.

Protecting packaged food from deterioration by foodborne germs during food processing and manufacturing is a significant consideration for food preservation. The AgNPs/MMT nanocomposite was, therefore, utilized for antibacterial purposes in food preservation and packaging. The antibacterial potential of the AgNPs/MMT nanocomposite was achieved using the agar (disc and well) diffusion techniques toward three types of respective G− species (*E. coli*, *P. aeruginosa*, and *Salmonella* sp.) and three types of respective G+ species (*S. aureus*, *L. monocytogenes*, and *B. cereus*) pathogens using four various concentrations (25, 50, 75, and 100 µg/mL) of a tested nanocomposite (Figure 5). Results revealed that the maximum ZOI was recorded, which indicated the lowest sensitivity, for *P. aeruginosa*, where the values of ZOI were 17, 22, 27, and 32 mm, respectively, with concentrations of 25, 50, 75, and 100 µg/mL. However, among the G+ species, *L. monocytogenes* exhibited the lowest ZOI. Data in Figure 5 illustrated that enlarged inhibitory activity correlated to a rise in AgNP level, possibly as a consequence of much more Ag getting released at a higher dosage. AgNPs were discovered to have comparable inhibitory activity on all microorganisms tested. Among the assigned three G− bacterial pathogens, *P. aeruginosa* displayed the greatest ZOI, followed by *Salmonella* and *E. coli*, exhibiting sensitiveness to the AgNPs/MMT.

On the other hand, G+ bacterial species were more unsusceptible to the tested nanocomposite. The internalization of the NPs and Ag^+^ ions inside the cells of *P. aeruginosa* was more significant than that of the other pathogens. In the current investigation, according to a few reports, G− bacteria have different cell wall constituents than G+ bacteria, which facilitate the ability for Ag^+^ ions produced by AgNPs to penetrate their cell wall. Because of the thickness and construction of their cell walls, G− bacteria were identified as being more effectively inhibited by AgNPs than G+ bacteria [35,36].

### 2.3. Estimation of MIC and MBC Values

The MIC and MBC values of the tested AgNPs/MMT nanocomposites toward the G− bacteria *E. coli*, *Salmonella*, and *P. aeruginosa*, as well as the G+ bacteria *S. aureus*, *L. monocytogenes*, and *B. cereus*, were appraised by testing different concentrations of the nanocomposite (15–100 µg/mL). The assessed MIC and MBC values of the tested AgNPs/MMT against the selected pathogens are shown in Table 3. The results displayed the MIC values of AgNPs/MMT against *E. coli*, *P. aeruginosa*, *Salmonella*, *S. aureus*, *L. monocytogenes*, and *B. cereus* were 30 ± 0.25, 15 ± 0.47, 30 ± 0.31, 45 ± 0.29, 75 ± 0.43, and 60 ± 0.53 µg/mL, respectively. Further, the MBC values were 45 ± 0.34, 30 ± 0.58, 45 ± 0.12, 60 ± 0.72, 75 ± 0.39, and 60 ± 0.28 µg/mL for *E. coli*, *P. aeruginosa*, *Salmonella*, *S. aureus*, *L. monocytogenes*, and *B. cereus*, respectively. The results indicated that the lowest concentration of AgNPs/MMT was observed for *P. aeruginosa*. These results are close to the results described by Sivanandy et al. [37], who attended that AgNPs could efficiently hinder the growth of *P. aeruginosa* at a relatively low dose. In addition to having a sizable and outstanding surface area that allows for broad interaction with bacteria, Ag NPs’ size makes it straightforward for them to penetrate the bacterial cytoplasm. This may be the cause of their superior antibacterial performance [38].

### 2.4. Dose- and Time-Dependent Killing Action

**Figure 6:**
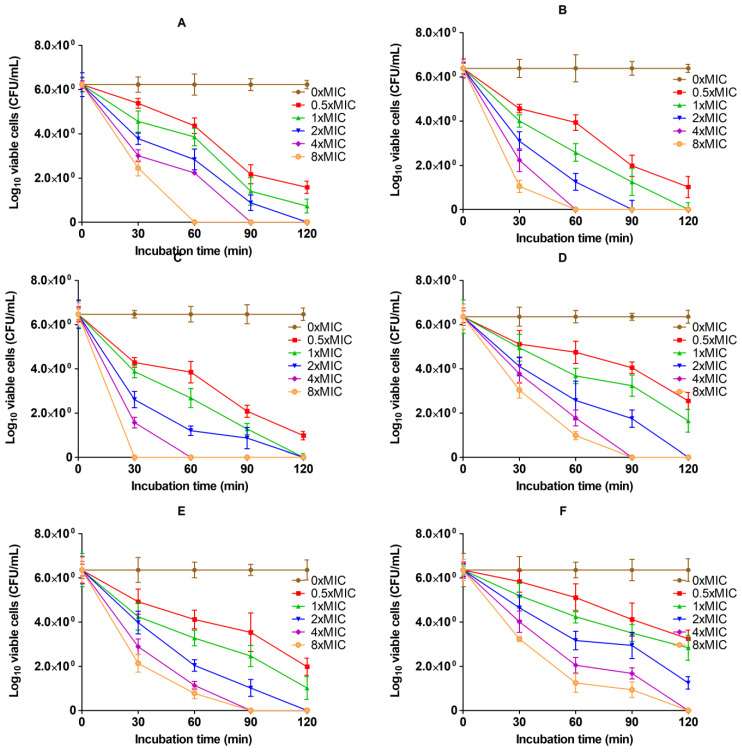
Time- and dose-killing plots of the AgNPs/MMT nanocomposite against (**A**) *E. coli*, (**B**) *Salmonella* spp., (**C**) *P. aeruginosa*, (**D**) *S. aureus*, (**E**) *L. monocytogenes*, and (**F**) *B. cereus* at various concentrations and time courses.

The results of the effect of dosage and time-dependent bactericidal activity of the AgNPs/MMT nanocomposite against the selected foodborne bacteria strains are shown in Figure 6. The results revealed that the viability of bacterial cells before and after exposure to the effective dose of AgNPs/MMT nanocomposite obviously diminished. The bactericidal activity of 8× MIC of AgNPs/MMT nanocomposite was efficacious against the specified G− pathogens, including *E. coli*, *Salmonella* spp., and *P. aeruginosa*; the diminishing in the cell densities was ≥6 Log CFU/mL (100%) after 30 min for *P. aeruginosa*, and 60 min of the incubation time course for *E. coli*, *Salmonella* spp. At the same time, G+ bacterial species such as *S. aureus*, *L. monocytogenes*, and *B. cereus* required a more extended time, about 90 min for *B. cereus* and 120 min for *S. aureus* and *L. monocytogenes*. For G− spoilage bacterial isolates, the potent bactericidal efficacy of the AgNPs/MMT nanocomposite for *E. coli* and *Salmonella* spp. was attained after 60, 90, and 120 min of incubation time at 8× MIC (8 × 30 μg/mL), 4× MIC (4 × 30 μg/mL), and 2× MIC (2 × 30 μg/mL); whereas for *P. aeruginosa*, the bacterial cells were eliminated after 120 min at 1× MIC (1 × 15 μg/mL) and 2× MIC (71 × 15 μg/mL), and after 30 min at 8× MIC (1 × 15 μg/mL). On another side, the antibacterial action of AgNPs/MMT nanocomposite against G− spoilage bacterial isolates and the vigorous bactericidal endpoint of the AgNPs/MMT nanocomposite for *S. aureus* and *L. monocytogenes* was achieved after 120 min of incubation time at 8× MIC (8 × 30 μg/mL) and 4× MIC (4 × 30 μg/mL). This demonstrates that AgNPs are ubiquitous bioactive compounds that have the same effect on all types of G− and G+ bacteria.

AgNPs can inhibit bacterial cell proliferation in a dose-dependent approach, as seen by the diminished survivability of microorganisms at greater its levels. These results also match the MIC values reported for each bacterial strain. A fundamental factor contributing to bacteria’s infectiousness is its higher incidence of multiplication [39]. Nevertheless, the bacteria’s reproduction cycle may be the best strategy for minimizing sustained contamination because AgNPs effectively stopped and eliminated the bacteria in a dose- and time-dependent approach, as demonstrated in the time-kill experiments. According to Zhang et al. (2016), relatively small AgNPs have more contact area than bigger ones; they might be more harmful to microorganisms and show excellent antimicrobial properties. In earlier research, 10 µg/mL of AgNPs prevented *E. coli* from growing in liquid Mueller–Hinton broth. Furthermore, it was determined that AgNPs’ MIC for *S. aureus* was 5.6 g/mL [40,41].

### 2.5. Change in Bacterial Cell Morphology and Antibacterial Mechanisms of AgNPs@MMT

**Figure 7:**
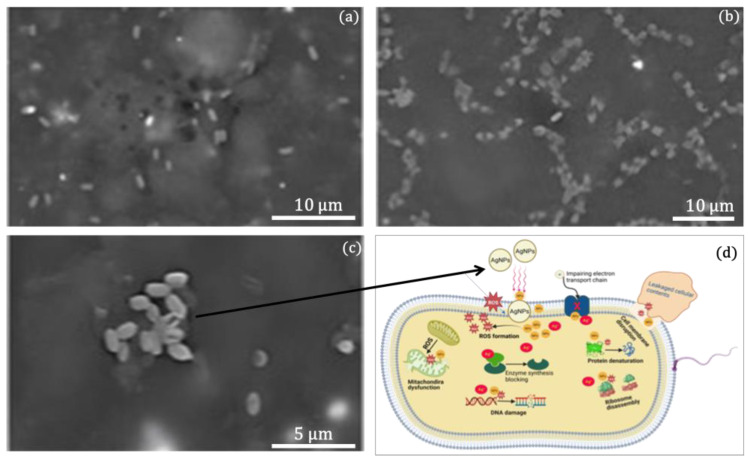
FESEM micrographs of (**a**) typical bacterial cell structure and (**b**,**c**) treated with AgNPs for 60 min. (**d**) A schematic illustration of the antimicrobial mechanism of AgNPs.

FESEM examination was employed to entirely investigate the morphological alterations that AgNPs/MMT nanocomposite caused in normal bacteria cells. Without being exposed to NPs, the test-control *E. coli* bacteria exhibited smooth, wholesome cells devoid of disruption (Figure 7a). Conversely, during 60 min of incubation, substantial changes in bacterial formation were observed in the AgNP-treated bacterial cells. Interestingly, bacterial cells treated with AgNPs displayed morphological deformation and abandoned their original morphological characteristics. Moreover, it was discovered that bacterial cells treated with AgNPs had destroyed cell walls and lacked membrane permeability (Figure 7b,c). The obtained outcomes are compatible with the results reported by Gopinath et al. [42], who revealed that the development of crumples and damage of the cell wall of bacterial cells treated with AgNPs were noticed.

As shown in Figure 7c, the proposed diagram of the antibacterial mechanism of AgNPs/MMT nanocomposites against normal bacterial cells is explained. Two main theories have been suggested concerning how AgNPs perform their antibacterial properties. These involve (i) an active engagement of the NPs with the bacterial cell membrane and (ii) the liberation of Ag^+^ ions [43]. The first theory postulates that AgNPs would adhere to the bacterial cell’s outer layer either by physical contact with sulfur-containing phosphoproteins in the cell wall or via electrostatic interactions between the positively charged NP ions and the negatively charged sites of the cell wall [44]. In the second one, the entrance of AgNPs into the cell might trigger the release of Ag^+^ ions and an upsurge in reactive oxygen species (ROS), which would then restrict the synthesis of enzymes, damaging the DNA and protein degradation and then ultimately causing bacterial cell death [45]. It must be highlighted that bacterial cells’ mitochondrial oxidative phosphorylation produces ROS inside the cells [46].

## 3. Materials and Methods

### 3.1. Chemicals

Throughout the current study, all chemicals and reagents were of high analytical grades. Montmorillonite (MMT) with a particle that is between 40–60 μm in size was supplied from Alfa Aesar GmbH & Co., Karlsruhe, Germany. Further, high-purity AgNO_3_ (purity of 99.98%, Merck, Darmstadt, Germany) was utilized as the silver predecessor. Meanwhile, the reducing agent (sodium borohydride; NaBH_4_, purity of 98.5%) was obtained from Sigma, Germany. All the standard solutions were prepared in distilled water.

### 3.2. Synthesis of AgNPs/MMT Nanocomposites

**Figure 8:**
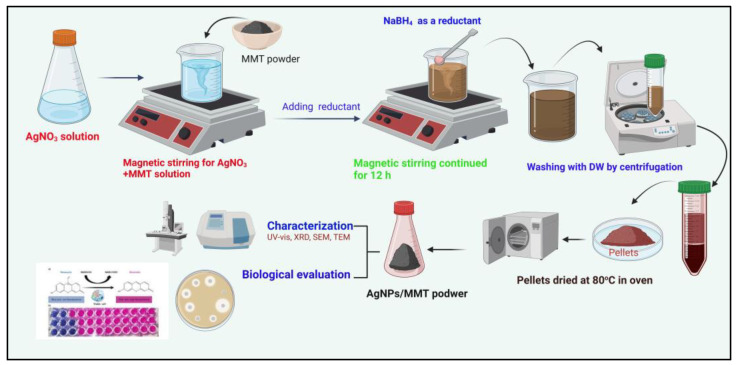
A schematic diagram for the synthesis of the AgNP/MMT nanocomposite.

According to the procedure previously reported by Shameli et al. [18], the AgNPs/MMT nanocomposite was successfully synthesized. Succinctly, 3.25 g of AgNO_3_ and 10 g of MMT were combined in the container containing distilled water (250 mL) and then positioned on a magnetic stirrer to be mixed overnight at room temperature (~25 °C). After that, 2.8 g of NaBH_4_ as a reducing agent was supplied into the suspension, and the prepared mixture was stirred for 12 h. The final preparation step was conducted by centrifuging the AgNPs/MMT mixture at 5000 rpm for 20 min. The Ag^+^ ion detritus was dismissed from the supernatant, and the pellets were harvested for washing and drying at 40 °C overnight (Figure 8).

### 3.3. Characterization Methods and Instruments 

The absorption spectrum of the reduced mixture of the AgNPs/MMT nanocomposite was established initially using UV–Vis spectroscopy. The absorption ranges were recorded between 200 nm and 700 nm using a spectrophotometer Jasco V-630, indiamart, Maharashtra, India. 

Transmission electron microscope. The morphological properties and particle dimensions Ag/NPs/MMT were explored using transmission electron microscopy (HRTEM, JEOL JEM-1011, Kyoto City, Japan). Size and dispersals of particles of sonicated dispersed suspension of the AgNPs/MMT nanocomposite were measured using a dynamic light scattering (DLS) instrument (PSS, Santa Barbara, CA, USA) and zeta potential (Nano-ZS, Malvern Instruments Ltd., London, UK). The fabricated Ag/MMT structures were observed using powder X-ray diffraction with a Diano X-ray diffractometer (Schimadzu 7000, Kyoto, Japan). The surface morphological features of the AgNPs/MMT were explored using a field emission scanning electron microscope (SEM, TESCAN FE-SEM MIRA3). 

### 3.4. Analysis of Food Product Samples by the Cultural Method

#### Food Sample Collection and Preparation

A total of 60 samples of arbitrarily packaged food were collected from food vendors, including supermarkets and convenience stores, and then taken directly to the laboratory for testing. The consistency of collected samples was uncooked and fully prepared, consisting of 15 samples of fresh cheese, 15 samples of fresh fish, and 15 samples of meat, fresh fish, and other food items. There were also 15 samples of fresh food of plant origin. For pre-enrichment, each collected sample’s weight (5 g) was blended by adding 25 mL of sterilized peptone water (PW), deposited in a Stomacher bag, and mashed for 5 min. 

### 3.5. Isolation and Identification of Foodborne Pathogens

Some particular foodborne bacterial pathogens, including *E. coli*, *Salmonella* spp., *P. aeruginosa*, *S. aureus*, *L. monocytogenes*, and *B. cereus*, respectively, were isolated by culturing upon specific media (Sigma-Alrdich, Burlington, MA, USA) such as Eosin methylene blue (EMB) agar, Bismuth sulfite agar, Acetamide agar, Modified (Twin Pack), Baird Parker agar, Al-Zoreky-Sandine *listeria* agar, and Bacillus differential agar. The essential steps of the workflow were a pre-enrichment step using PW as a non-selective broth medium, a pour plate methods step using selective agar media, and the isolation of the presumed distinctive colonies [47]. A single identical colony of each bacterial isolate comprising *E. coli*, *P. aeruginosa*, *Salmonella* spp., *S. aureus*, *L. monocytogenes*, and *B. cereus* was picked up from the agar media for identification. The bacterial cultures of the isolates were maintained and preserved at −80 °C on a nutrient agar medium [48]. The phenotype microarray instrument was employed for phenotyping identification to verify the isolates. A small part of the typical colony was taken and transferred into an inoculating fluid A” tube. All implanted fluid tubes were placed in an incubator for 18–24 h at 37 °C. By multichannel micropipette, 100 µL of fluid was transferred to each MicroPlate for each isolate before being maintained for 20 h at 37 °C. The microplates were scanned in the MicroStation semi-automated reader [49].

### 3.6. Antimicrobial Evaluation

#### Stock Solutions

In 5 mL of dimethyl sulfoxide (DMSO), an appropriate weight (100 mg) of the AgNPs/MMT nanocomposite was dissolved. For subsequent research, several nanocomposite concentrations were created. A positive control medication was employed: ciprofloxacin (µL).

### 3.7. Inhibition Study Using Agar Diffusion Method

The bactericidal effect of the chemically prepared AgNPs/MMT nanocomposite was appraised toward the six foodborne bacterial isolates, including *E. coli*, *P. aeruginosa*, and *Salmonella* spp., as a model for gram-negative (G−) species, and *S. aureus*, *L. monocytogenes*, and *B. cereus* were a model for gram-positive (G+) species. According to CLSI [50], The bactericidal action of the nanocomposite against certain bacterial species was achieved using the Kirby–Bauer diffusion sensitivity assay [51]. The bacterial 24 h cultures were disseminated over the surface of the Mueller–Hinton agar (MHA) (HiMedia, India). The disc (6 mm) was impregnated, and the well was occupied with 50 µL of four various concentrations of the AgNPs/MMT solution (25, 50, 75, and 100 µg/mL). The soaked discs were then positioned on the surface of the agar plate and incubated overnight at 37 °C. The experimental zone of inhibition (ZOI) was measured after 24 h of incubation. The ZOI created near the discs and wells, which indicated the bactericidal action of the AgNPs/MMT, was measured in mm [52].

### 3.8. MIC and MBC Estimation

The MIC was measured using a resazurin-based experiment using several AgNPs/MMT solution concentrations and an adjustable bacterial load (10^7^ CFU/mL). The MIC assessment was completed using two 96-well microplates. Mueller–Hinton broth (MHB), 190 mL, and 10 mL of each pathogen species were added to the first plate’s first six columns as a positive control. The AgNPs/MMT nanocomposite was prepared by diluting in the other six columns of the first plate and the second plate, with concentrations that ranged from 15 to 100 µg/mL (15, 30, 45, 60, 75, and 100) and 10 µL of each bacterial suspension. Three columns in the third plate were utilized as a negative control and contained 150 µL of MHB. During 24 h of incubation time, 15 µL of a 0.02% fresh resazurin solution was introduced into each microtiter plate well. The plates were then stored in the incubator at 37 °C for an extended 4 h. The estimated MIC value is defined as the smallest dosage of tested nanocomposite that still prevented bacterial evolution [53]. A further definition of the MBC is defined as the smallest dosage of an effective antibacterial agent required to destroy all microorganisms. By spreading 100 µL of the bacteria culture from the separate well in the microtitre plates into the MHA agar, the MBC values test was calculated. The implanted plates were incubated for 24 h at 37 °C. MBC value was the smallest dosage on the plate without any observable bacterial growths [54].

### 3.9. Dose- and Time-Dependent Killing Effect Assay

A time-kill experiment was performed using the fresh bacterial cells of the investigated strains to establish the appropriate exposure time. Apparently, there were about 10^6^ CFU/mL of bacterial strains in the inoculum suspension. The stock solution of AgNPs/MMT was diluted with the MHB medium containing inoculum to generate different concentrations (0× MIC, 0.5× MIC, 1× MIC, 2× MIC, 4× MIC, and 8× MIC). The mixture (10 mL final volume) was agitated at 200 rpm and maintained at 37 °C. A total of 100 µL of aliquots were pulled out at pre-determined intervals (0, 30, 60, 90, and 120 min), transported, sequentially diluted with 1M phosphate-buffered saline (PBS), and then mounted onto the MHA. The number of colonies was counted, and represented as CFU/mL [55]. 

### 3.10. Morphological Change in Bacterial Cells

FESEM (FE-SEM, a Quanta FEG 250, Czech Republic) was employed to investigate the differences in bacterial cell structure before and after exposure to the effective concentration (MIC) of the AgNPs/MMT solution. Subsequently, the bacterial cells were fixed in 2.5% glutaraldehyde for 4 h. After that, the fixed cells were washed thoroughly with PBS before being sequentially dehydrated with ethanol (30%, 40%, 50%, 70%, 80%, 90%, and 100% for 15 min per phase). The dried cell was then placed on stubs and sprayed with gold film in a sputter coater [42].

### 3.11. Statistical Analysis

Independent examinations were executed in duplicate. A graph of the log CFU/mL vs. time was displayed. The one-way ANOVA strategy was implemented to evaluate the outcomes of the research. For the research investigations, a *p*-value of 0.05 was used as the significance criterion.

## 4. Conclusions

The chemically synthesized Ag NPs/MMT nanocomposite was effectively fabricated from the AgNO_3_/MMT suspension using NaBH_4_ at an ambient temperature. A few NPs were agglomerated between adjacent MMT membranous layers, but the majority of AgNPs, as suggested by the XRD data and displayed in TEM appearance, were only present at the outermost MMT layers. Further, the XRD data verified that the silver crystal’s crystallographic surfaces were fcc. The peak characteristic of the SPR bond in AgNPs was realized in the UV–visible absorption spectra at 416 nm. The exterior structure of MMT and Ag/MMT is portrayed by multilayered interfaces with sizable flakes in SEM images, with no discernible morphological differences among them. The phenotyping method was employed to identify six foodborne bacterial pathogens isolated from packaged food samples. Various doses of Ag/MMT were applied to examine their bactericidal effect on the isolated species using agar and broth experiments. The outcomes showed vigorous antibacterial activity against both G− and G+ bacteria. It is clear from the toxicity assay that the estimated MIC value was risk-free and biocompatible.

## Figures and Tables

**Table 2:** Relative abundance of some foodborne bacteria isolates collected from different food samples and confirmed by Biolog phenotype Microarray.

Bacterial Isolates	Meat		Fish		Cheese		Vegetables		Total Samples	
	+/No. of Isolates	%	+/No. of Isolates	%	+/No. of Isolates	%	+/No. of Isolates	%	+/Total No. of Isolates	%
*E. coli*	9/15	60	11/15	73.3	12/15	80	11/15	73.3	43/60	71.6
*Salmonella* spp.	11/15	73.3	12/15	80	8/15	53.3	10/15	66.6	41/60	68.3
*P. aureginosa*	10/15	66.6	9/15	60	7/15	46.6	5/15	33.3	31/60	51.6
*S. aureus*	10/15	66.6	8/15	53.3	5/15	33.3	6/15	40	29/60	48.3
*L. moncytogenes*	8/15	53.3	5/15	33.3	11/15	73.3	4/15	26.6	28/60	46.6
*B. cereus*	8/15	53.3	4/15	26.6	6/15	40	3/15	20	21/60	35

**Table 3:** Estimated values of the MIC and MBC of the AgNPs/MMT against selected foodborne bacterial pathogens (G− and G+ bacteria).

Selected Bacterial Strains		Microdilution Assay (µg/mL)	
		MIC	MBC
G− bacterial strains	*E. coli*	30 ± 0.25	45 ± 0.34
	*P. aeruginosa*	15 ± 0.47	30 ± 0.58
	*Salmonella* sp.	30 ± 0.31	45 ± 0.12
G+ bacterial strains	*S. aureus*	45 ± 0.29	60 ± 0.72
	*L. monocytogenes*	75 ± 0.43	75 ± 0.39
	*B. cereus*	60 ± 0.53	60 ± 0.28

## Data Availability

Not applicable.

## Supplementary Materials

The following supporting information can be downloaded at: https://www.mdpi.com/article/10.3390/molecules28093699/s1, Figure S1: Illustration of design of phenotype 96-well the microPlate; and Figure S2: Phenotypic features of verified (a) *E. coli*, (b) *Salmonella* spp., (c) *Pseudomonas aeruginosa*, (d) *Listeria monocytogenes*, (e) *Staphylococcus aureus*, and (f) *Bacillus cereus*.
