# Supplementary material for: Facile Synthesis, Characterization, and Antimicrobial Assessment of a Silver/Montmorillonite Nanocomposite as an Effective Antiseptic against Foodborne Pathogens for Promising Food Protection
Source: Molecules. 2023 Apr 25;28(9):3699. doi: 10.3390/molecules28093699 (PMC10180218; doi:10.3390/molecules28093699)
Supplement: Supplementary file 1 [file molecules-28-03699-s001.zip › molecules-2333908-supplementary.pdf]

Facile synthesis, characterization, and antimicrobial assessment of silver/montmorillonite nanocomposite as an effective antiseptic against foodborne pathogens for promising food protection

Mohsen M. El-Sherbiny [1,*], Reny P. Devassy [1], Mohamed E. El-Hefnawy [2], Soha T. Al-Goul [2], Mohamed I. Orif [3] and Mohamed H. El-Newehy [4,5,*]

[1] Department of Marine Biology, Faculty of Marine Sciences, King Abdulaziz University, Jeddah 21589, Saudi Arabia; renydevassy@gmail.com

[2] Department of Chemistry, Rabigh College of Sciences and Arts, King Abdulaziz University, Jeddah 21589, Saudi Arabia; malhefnawy@kau.edu.sa (M.E.E.-H.); salgoul@kau.edu.sa (S.T.A.-G.)

[3] Department of Marine Chemistry, Faculty of Marine Sciences, King Abdulaziz University, Jeddah 21589, Saudi Arabia; mioraif@kau.edu.sa

[4] Department of Chemistry, Faculty of Science, Tanta University, Tanta 31527, Egypt

[5] Department of Chemistry, College of Science, King Saud University, Riyadh 11451, Saudi Arabia

* Correspondence: ooomar@kau.edu.sa (M.M.E.-S.); melnewehy@ksu.edu.sa (M.H.E.-N.)

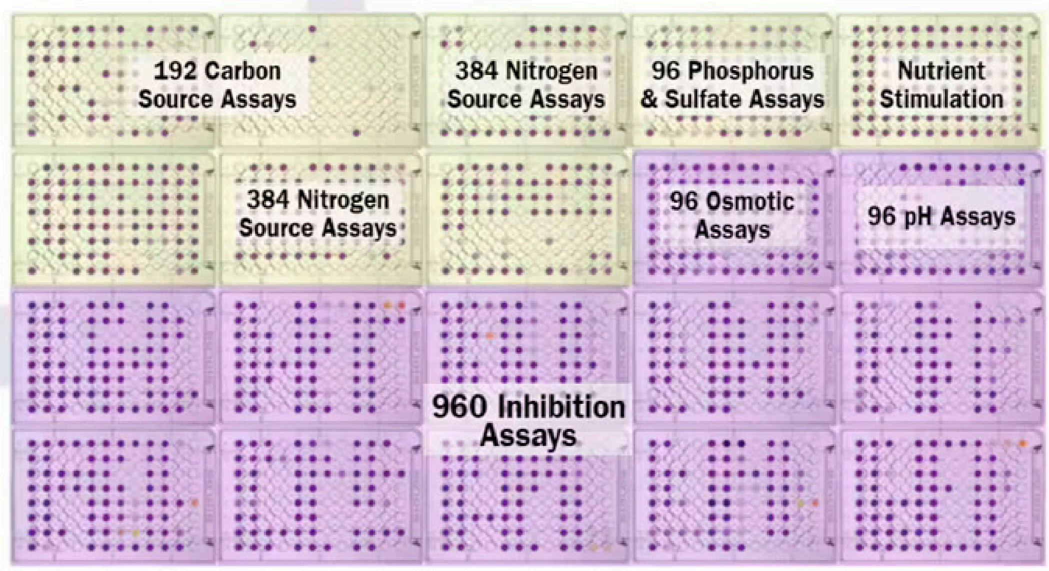

Figure S1. Illustration of design of phenotype 96-well the microPlate.

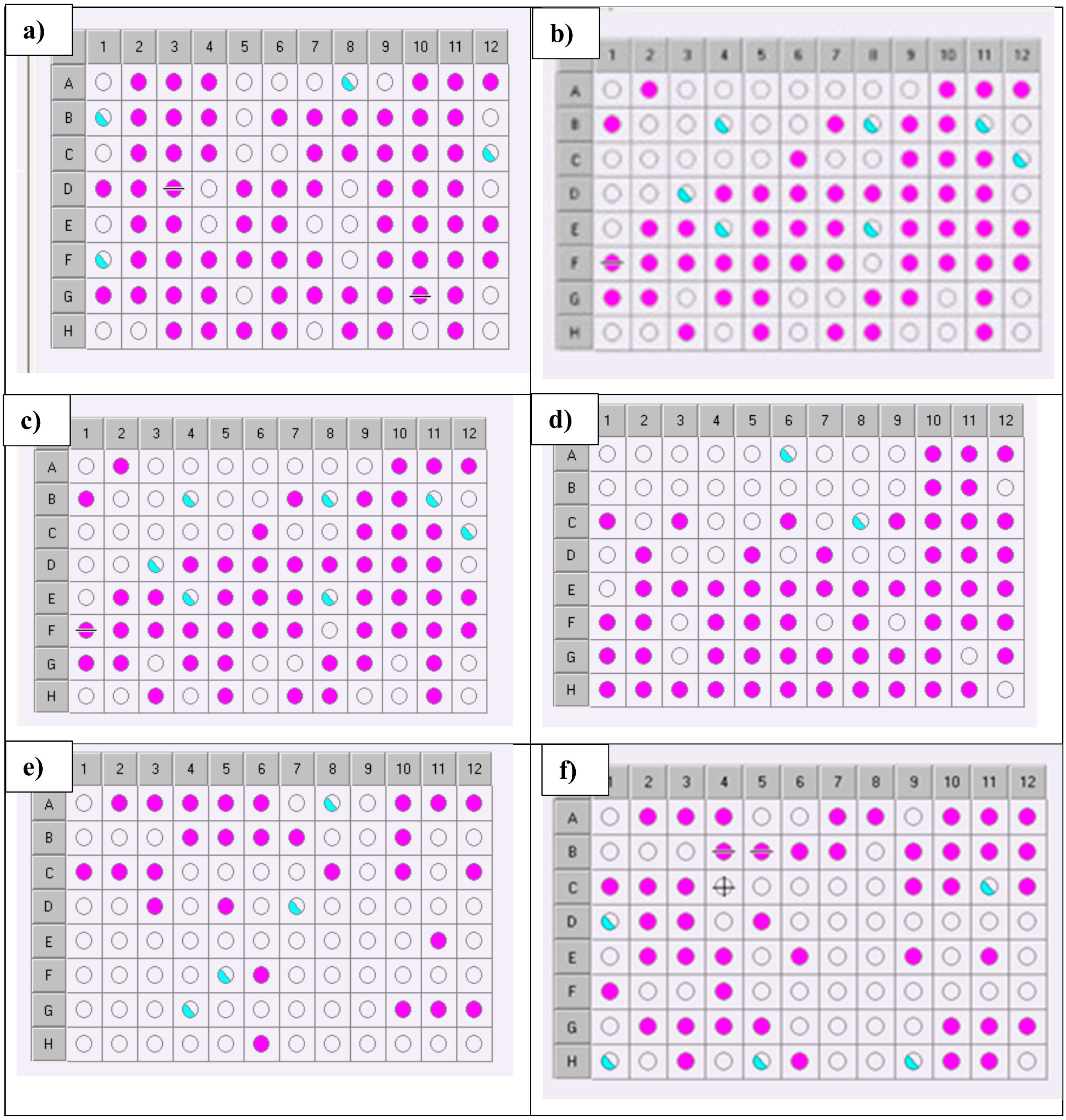

Figure S2. Phenotypic features of verified a) *E. coli*, c) *Salmonella* spp., c) *Pseudomonas aeruginosa*, d) *Listeria monocytogenes*, e) *Staphylococcus aureus*, and f) *Bacillus cereus*.
